# Supplementary material for: A high-throughput newborn screening approach for SCID, SMA, and SCD combining multiplex qPCR and tandem mass spectrometry
Source: PLoS One. 2023 Mar 10;18(3):e0283024. doi: 10.1371/journal.pone.0283024 (PMC10004496; doi:10.1371/journal.pone.0283024)
Supplement: S1 Fig — (A) For the qPCR-based approach for SCID, SMA and 1st-tier SCD newborn screening. (B) For the MS/MS-based differentiation for 2nd-tier SCD newborn screening. This figure is accompanying the decision workflow depicted in Fig 2 of the main manuscript; prepared with Inkscape (1.2.2). (PDF) [file pone.0283024.s001.pdf]

S1 Figure. Tesorero et al. 2023

A.

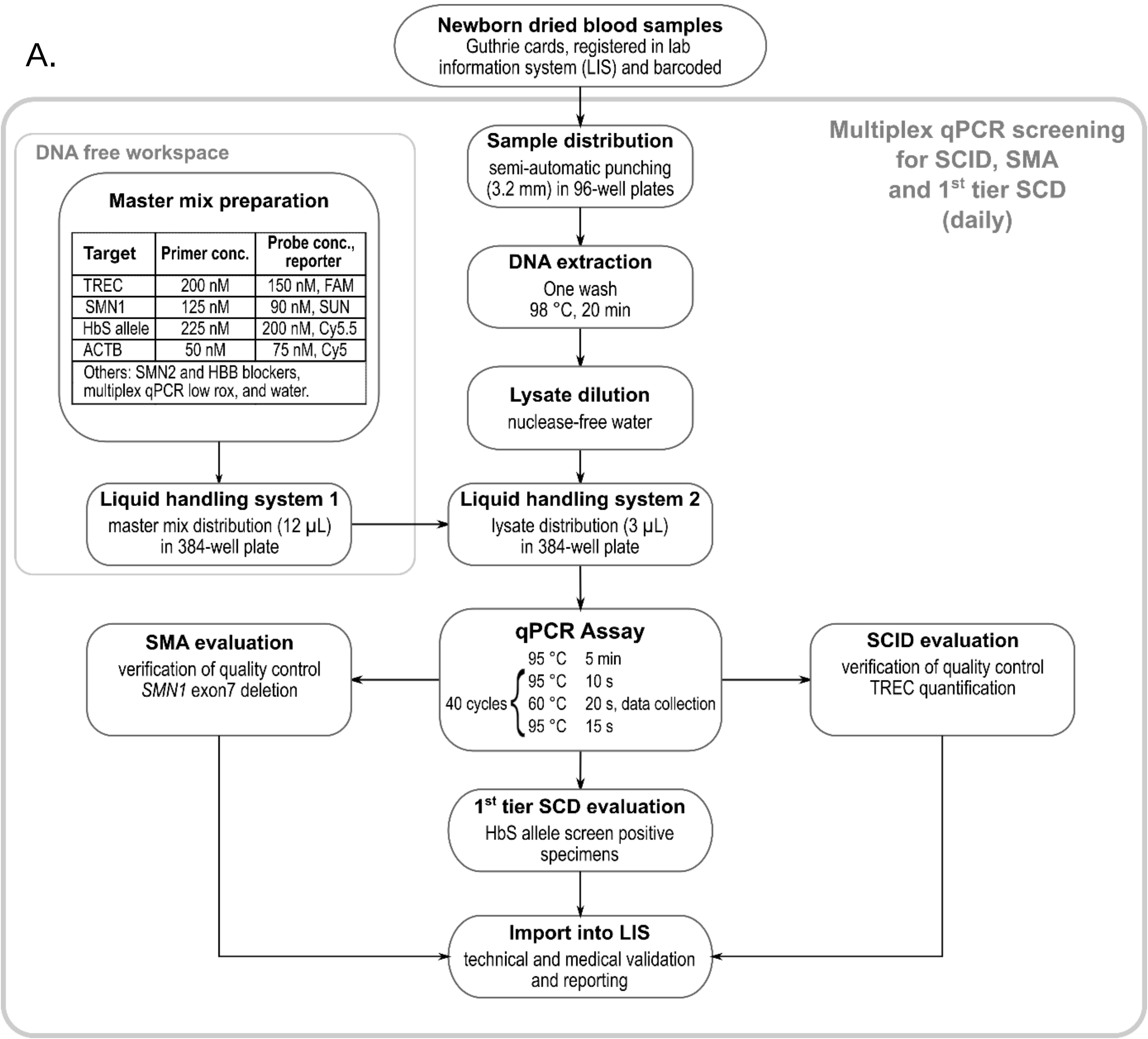

B.

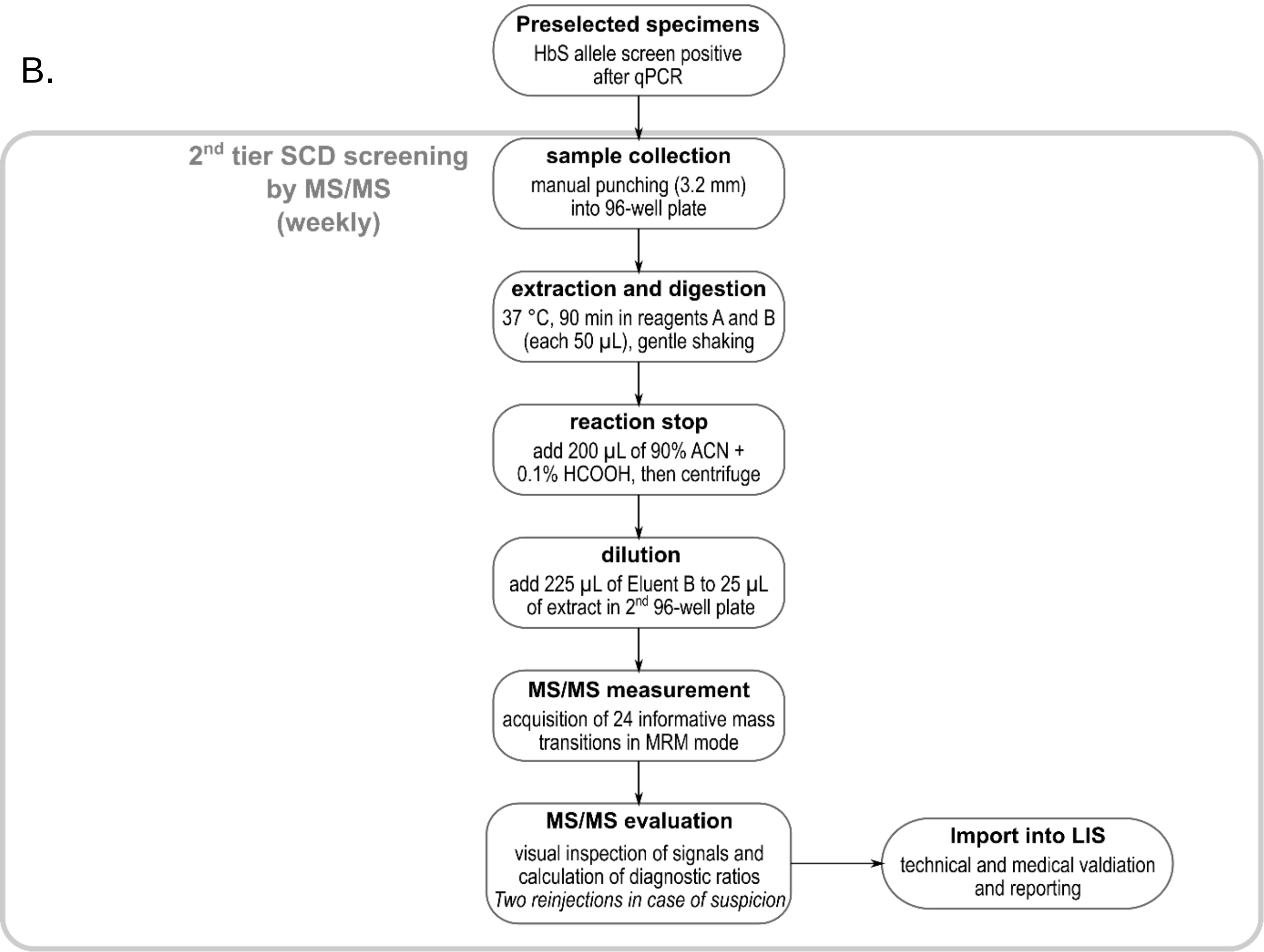

S1 Figure. Workflow representing the tasks for sample preparation and analysis. (A) For the qPCR-based approach for SCID, SMA and 1st-tier SCD newborn screening. (B) For the MS/MS-based differentiation for 2nd-tier SCD newborn screening. This figure is accompanying the decision workflow depicted in Fig 2 of the main manuscript; prepared with Inkscape (1.2.2).
