# Supplementary material for: A high-throughput newborn screening approach for SCID, SMA, and SCD combining multiplex qPCR and tandem mass spectrometry
Source: PLoS One. 2023 Mar 10;18(3):e0283024. doi: 10.1371/journal.pone.0283024 (PMC10004496; doi:10.1371/journal.pone.0283024)
Supplement: S2 Fig — All spectra were recorded in ‘Multi Channel Analysis’ mode Acquisition during 30 s with cycle time set to automatic and 2.0 s scan duration. The spectra were obtained by infusing solutions of the peptide (1.0 μg/mL) dissolved in an acetonitrile-based (solid line) and methanol-based (dotted) mobile phase. (PDF) [file pone.0283024.s002.pdf]

S2 Figure. Tesorero et al. 2023

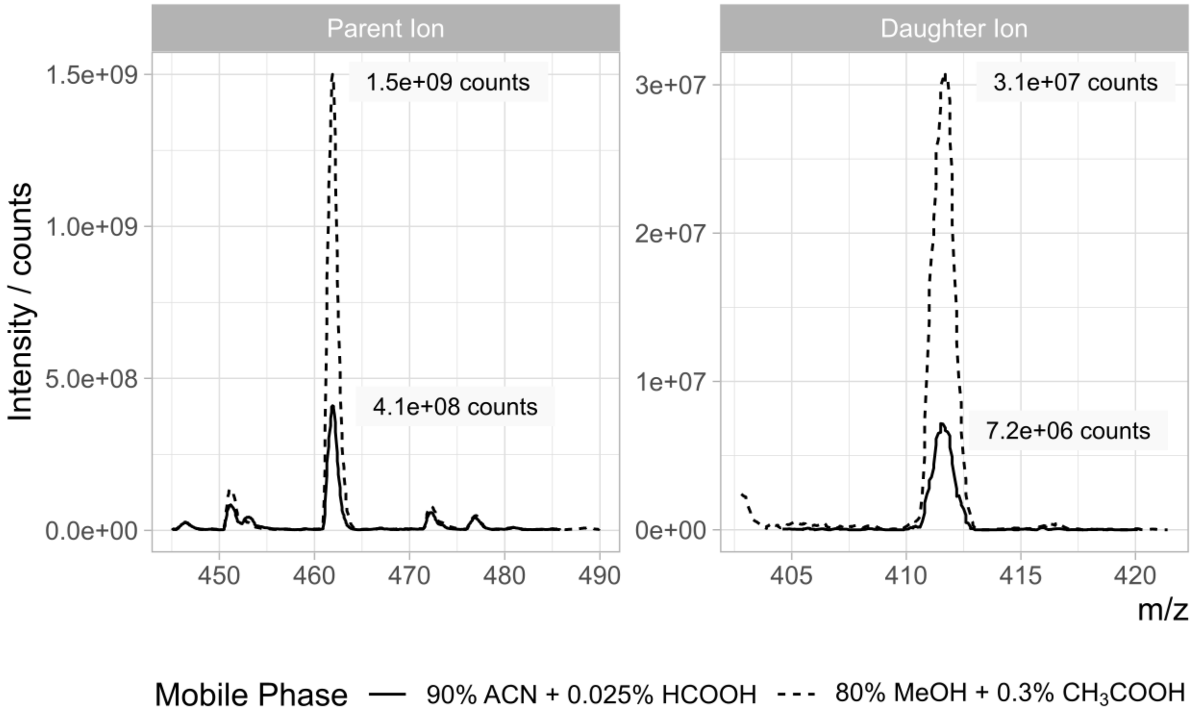

S2 Figure. Mass spectra for the HbS bT1 peptide (Parent Ion) acquired in MS1 scan and its y7-fragment (daughter Ion) acquired in MS2 scan. All spectra were recorded in 'Multi Channel Analysis' mode acquisition during 30 s with automatic cycle time adjustment and 2.0 s scan duration. The spectra were obtained by infusing solutions of the peptide (1.0 µg/mL) dissolved in an acetonitrile-based (solid line) and methanol-based (dotted) mobile phase.
