# Supplementary material for: A high-throughput newborn screening approach for SCID, SMA, and SCD combining multiplex qPCR and tandem mass spectrometry
Source: PLoS One. 2023 Mar 10;18(3):e0283024. doi: 10.1371/journal.pone.0283024 (PMC10004496; doi:10.1371/journal.pone.0283024)
Supplement: S3 Fig — The signal for the HbS allele is not amplified in any of the samples. The corresponding MS/MS results are shown in S4 Table. (PDF) [file pone.0283024.s003.pdf]

S3 Figure. Tesorero et al. 2023

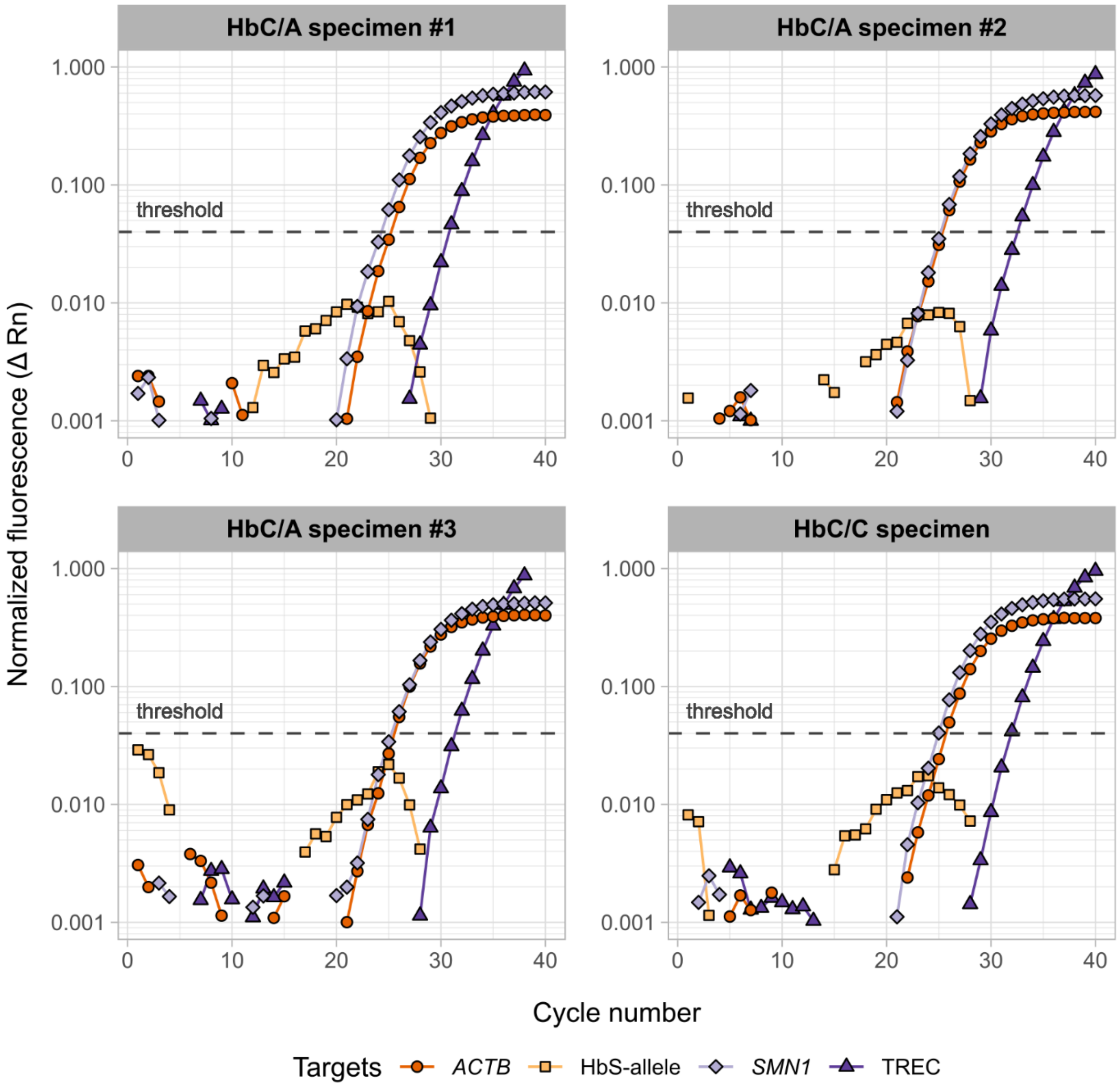

S3 Figure. Amplification plots for four exemplary HbC-containing specimens devoid of HbS (three HbC/A and one HbC/C). The signal for the HbS allele is not amplified in any of the samples. The corresponding MS/MS results are shown in S4 Table.
