# Supplementary material for: A high-throughput newborn screening approach for SCID, SMA, and SCD combining multiplex qPCR and tandem mass spectrometry
Source: PLoS One. 2023 Mar 10;18(3):e0283024. doi: 10.1371/journal.pone.0283024 (PMC10004496; doi:10.1371/journal.pone.0283024)
Supplement: S1 Table — Bovine RBC was 1:2 diluted with TE buffer pH 8.0 and different synthetic dsDNA (gBlocks, IDT) were used to spike the different QCs. RBC: Red blood cells. (PDF) [file pone.0283024.s004.pdf]

S1 Table. Quality controls (QCs) composition used in the qPCR assay. Bovine RBC was 1:2 diluted with TE buffer pH 8.0 and different synthetic dsDNA (gBlocks, IDT) were used to spike the different QCs. RBC: red blood cells.

Quality Control	Purpose	Content
**QC1**	Negative control:<br>No synthetic dsDNA added	• Bovine RBC<br>• 1x TE Buffer pH 8.0
**QC2**	Positive control:<br>Screening negative sample	• Bovine RBC<br>• 1x TE Buffer pH 8.0<br>• *ACTB*<br>• *SMN1* (Exon-7)<br>• *HBB* (CD-6)<br>• sj-TREC
**QC3**	Positive control:<br>Screening positive sample	• Bovine RBC<br>• 1x TE Buffer pH 8.0<br>• *ACTB*<br>• *SMN2* (Exon-7, c.840C>T)<br>• HbS allele (*HBB*: c.20A>T)
