# Supplementary material for: A high-throughput newborn screening approach for SCID, SMA, and SCD combining multiplex qPCR and tandem mass spectrometry
Source: PLoS One. 2023 Mar 10;18(3):e0283024. doi: 10.1371/journal.pone.0283024 (PMC10004496; doi:10.1371/journal.pone.0283024)
Supplement: S2 Table — (PDF) [file pone.0283024.s005.pdf]

S2 Table. Targeted hemoglobin peptides, assigned mass transitions, and optimized mass spectrometric parameters used in the 2nd tier instrumental analysis.

Target	Peptides and fragments	Parent m/z /Da	Daughter m/z /Da	Collision energy / V
**HbS**	bT1 y4	461.8	472.3	21
	bT1 y7	461.8	412.3	18
**HbC**	bT1 b3	347.7	350.3	18
	bT1 y5	347.7	298.2	15
**HbD^{punj}**	bT13 b2	689.4	276.1	18
	bT13 b3	689.4	377.2	21
**HbO^{arab}**	bT13 y9	625.4	1001.6	18
	bT13 y9	625.4	501.3	18
**HbE**	bT3 y6	458.8	604.3	18
	bT3 y7	458.8	703.4	18
**HbF**	gT5 y4	995.47	415.2	24
	gT2 y6	488.8	691.4	18
	gT2 y7	488.8	804.5	18
**HbLep**	dT2 y6	480.3	688.4	18
	dT2 y3	480.3	390.2	21
**HbA2**	dT14 y9	721.34	532.8	18
**HbA**	bT1 y4	476.85	502.2	24
	bT1 y7	476.85	427.3	18
	bT13 b2	689.9	277.1	21
	bT13 b3	689.9	378.2	18
	bT13 y9	689.9	1001.5	21
	bT3 y9	657.9	887.5	21
	bT3 y8	657.9	758.2	21
	bT2 y6	466.76	675.4	18
**IS digested**		465.8	480.3	21
**IS intact**		509.6	350.4	18
