# Supplementary material for: A high-throughput newborn screening approach for SCID, SMA, and SCD combining multiplex qPCR and tandem mass spectrometry
Source: PLoS One. 2023 Mar 10;18(3):e0283024. doi: 10.1371/journal.pone.0283024 (PMC10004496; doi:10.1371/journal.pone.0283024)
Supplement: S3 Table — (PDF) [file pone.0283024.s006.pdf]

S3 Table. Diagnostic ratios used in the 2nd tier MS/MS method, the mass transitions for their calculation, and the action values applied.

Ratio	Used mass transitions to calculate	Cutoff
IS-Ratio	IS digested / IS intact	
HbS/HbA_1	HbS_bT1_y4_1 / HbA_bT1_y4_1	2,67
HbS/HbA_2	HbS_bT1_y7_2 / HbA_bT1_y7_2	3,17
HbC/HbA_1	HbC_bT1_b3_1 / HbA_bT1_y4_1	1,0
HbC/HbA_2	HbC_bT1_y5_2 / HbA_bT1_y7_2	1,0
HbE/HbA_1	H bE_bT3_y6_1 / HbA_bT3_y9_1	0,19
HbE/HbA_2	HbE_bT3_y7_1 / HbA_bT3_y8_1	0,19
HbD^{punj}/HbA_1	HbD^{punj}_bT13_b2_1 / HbA_bT13_b2_1	1,0
HbD^{punj}/HbA_2	HbD^{punj}_bT13_b3_1 / HbA_bT13_b3_1	1,0
HbO^{arab}/HbA_1	HbO^{arab}_bT13_y9_1 / HbA_bT13_y9_1	0,6
HbLep/HbA_1	HbLep dT2 y6 / HbA bT2 y6	
HbLep/HbA_2	HbLep dT2 y3 / HbA bT2 y6	
HbF/HbA_1	HbF_gT2_y06 / HbA_bT2_y06_1	
HbF/HbA_2	HbF_gT5_y04 / HbA_bT3_y8_1	
HbA/HbF_1	HbA_bT1_y7_2 / HbF_gT2_y06	
HbA/HbF_2	HbA_bT3_y8_1 / HbF_gT2_y06	
