# Supplementary material for: A high-throughput newborn screening approach for SCID, SMA, and SCD combining multiplex qPCR and tandem mass spectrometry
Source: PLoS One. 2023 Mar 10;18(3):e0283024. doi: 10.1371/journal.pone.0283024 (PMC10004496; doi:10.1371/journal.pone.0283024)
Supplement: S4 Table — (PDF) [file pone.0283024.s007.pdf]

S4 Table. Diagnostic ratios obtained with the MS/MS assay for the four HbC-containing specimens corresponding to the amplification plots depicted in S3 Figure.

		Results			
**Ratio**	**Cutoff**	**HbC/A specimen #1**	**HbC/A specimen #2**	**HbC/A specimen #3**	**HbC/C specimen**
HbS/HbA_1	2,67	0.0006	0.0011	0.0004	2.0043
HbS/HbA_2	3,17	0.0005	0.0007	0.0067	0.0061
**HbC/HbA_1**	1,0	**1.7474**	**2.0168**	**2.1743**	**2297.0952**
**HbC/HbA_2**	1,0	**10.2245**	**11.5384**	**10.751**	**104.5898**
HbE/HbA_1	0,19	0.0037	0.0092	0.0003	0.0001
HbE/HbA_2	0,19	0.0931	0.0417	0.0032	0.4258
HbD^{punj}/HbA_1	1,0	0.0005	0.0007	0.0005	0.0052
HbD^{punj}/HbA_2	1,0	0.0002	0.001	0.0002	0.0006
HbO^{arab}/HbA_1	0,6	0.0203	0.0096	0.0243	0.0005
HbF/HbA_1		0.9399	0.9445	0.3758	2.1963
HbF/HbA_2		0.6409	0.4629	0.2087	1.0238
HbA/HbF_1		0.3014	0.2799	0.6443	0.017
HbA/HbF_2		0.4	0.4813	0.9356	0.1587
